# Mammographic tumour appearance is related to clinicopathological factors and surrogate molecular breast cancer subtype

**DOI:** 10.1038/s41598-020-77053-7

**Published:** 2020-11-30

**Authors:** Li Sturesdotter, Malte Sandsveden, Kristin Johnson, Anna-Maria Larsson, Sophia Zackrisson, Hanna Sartor

**Affiliations:** 1 Department of Translational Medicine, Diagnostic Radiology, Lund University, Lund, Sweden; 2 Department of Medical Imaging and Physiology, Skåne University Hospital, Lund/Malmö, Sweden; 3 Department of Clinical Sciences Malmö, Surgery, Lund University, Lund, Sweden; 4 Department of Surgery, Skåne University Hospital, Malmö, Sweden; 5 Department of Clinical Sciences Lund, Division of Oncology, Lund University, Lund, Sweden; 6 Department of Hematology, Oncology and Radiation Physics, Skåne University Hospital, Lund, Sweden

**Keywords:** Breast cancer, Breast cancer, Cancer imaging, Tumour biomarkers, Medical imaging, Epidemiology

## Abstract

Mammographic tumour appearance may provide prognostic useful information. For example, spiculation indicates invasiveness, but also better survival compared to tumours with other appearances. We aimed to study the relationship between mammographic tumour appearance and established clinicopathological factors, including surrogate molecular breast cancer subtypes, in the large Malmö Diet and Cancer Study. A total of 1116 women with invasive breast cancer, diagnosed between 1991 and 2014, were included. Mammographic tumour appearance in relation to status for oestrogen receptor (ER), progesterone receptor (PR), human epidermal growth factor receptor 2, histological grade, Ki67 and molecular subtype was analysed using various regression models. All models were adjusted for relevant confounders, including breast density, which can affect mammographic appearance. The results consistently showed that spiculated tumours are indicative of favourable characteristics, as they are more likely to be ER and PR positive, and more often exhibit lower histological grade and lower Ki67 expression. Furthermore, spiculated tumours tend to be of luminal A-like subtype, which is associated with a good prognosis. The establishment of associations between mammographic tumour appearance and clinico­pathological factors may aid in characterizing breast cancer at an earlier stage. This could contribute to more individualized breast cancer treatment in the future.

## Introduction

Mammography plays a fundamental role in breast cancer screening and diagnosis^1^. The images can provide crucial information, although their full potential has not yet been utilized in clinical practice. The mammographic appearance of breast lesions, both benign and malignant, corresponds to histopathological diagnoses, which can be predicted with varying certainty. For example, a spiculated (or stellate) lesion has a high positive predictive value for malignancy, often around 90%^2^, whereas a mass with a circumscribed contour most often indicates a benign lesion (e.g. fibroadenoma, cyst or hamartoma)^3^. However, a circumscribed mass can also be an invasive carcinoma without surrounding stromal reaction^3^, making diagnosis based on imaging alone unreliable. Several previous studies have been performed on the relation between the mammographic appearance of malignant tumours and diagnosis and survival. For example, tumours with casting-type calcifications are associated with a worse prognosis^4^. On the other hand, spiculation indicates invasiveness^5^, but at the same time a better survival rate than in those breast tumours with other mammographic appearances^6^. Breast density is also known to affect mammographic appearance^7^. Knowledge on clinicopathological factors such as tumour size, histological type, hormonal receptor status and axillary lymph node involvement is essential in breast cancer staging and the prediction of prognosis^8^. Our research group has previously studied mammographic tumour appearance in relation to invasiveness, tumour size and axillary lymph node involvement within a smaller subset of the same cohort used in this study, and found an association between spiculation and invasiveness, regardless of breast density^5^.

In addition to clinicopathological factors, surrogate molecular subtypes aid in the prediction of prognosis and the choice of appropriate treatment^8^. It has been suggested in the literature that the presence of calcifications on mammography is more common in the human epidermal growth factor receptor 2 amplified (HER2+) subtype^9,10^, while triple-negative breast cancer (TNBC) is more likely to present as a mass^10^. Few previous studies have, however, investigated the combination of the information available from mammographic appearance, clinicopathological factors, including molecular subtypes, and breast density. The aim of the present study was thus to investigate the relationship between mammographic tumour appearance and clinicopathological factors, including surrogate molecular subtypes, in the large cohort of incident breast cancer cases within the Malmö Diet and Cancer Study.

## Methods and material

### Study population

Data were collected from the Malmö Diet and Cancer Study (MDCS), a large population-based prospective cohort study, in which 28,098 individuals were enrolled from 1991 to 1996, of which 17,035 were women^11^. Inhabitants aged 44–74 years were recruited from the city of Malmö in the south of Sweden. The original intent of the study was to investigate possible associations between diet and cancer. At the baseline examination, anthropometric variables (blood pressure, height, weight, lean body mass and body fat mass) and blood samples were collected, and a comprehensive questionnaire was filled in^12^. The MDCS is updated regularly with information on new cancer cases through data collection from national registers: the Swedish Cause of Death Register, the Swedish Cancer Register, and the Regional Tumour Register for Southern Sweden^5^. Women with prevalent breast cancer at baseline or a history of breast cancer (n = 572) were excluded. A total of 1242 women in the cohort were diagnosed with incident breast cancer between 1991 and 2014. After the exclusion of carcinoma in situ (n = 105) and bilateral breast tumours (n = 21), 1116 women with invasive breast cancer remained eligible for the present study. Informed consent was obtained at the baseline examination. The Ethics Committee at Lund University approved this study (Official Records Nos. 652/2005 and 166/2007). The study was carried out in accordance with the declaration of Helsinki.

### Mammography

A protocol was set up to extract information from the original mammography report at the time of cancer diagnosis. This protocol had several variables, including mammographic tumour appearance, breast density and mode of cancer detection^5^. All screening mammograms were double read by two breast radiologists, but the diagnostic imaging during follow-up assessment was performed by one breast radiologist. Mammograms of clinically detected cancers were single read by one breast radiologist. When any of the information was lacking in the original mammography report, the mammograms were re-read by an experienced breast radiologist (SZ and/or HS).

#### Mammographic tumour appearance

**Figure 1:**
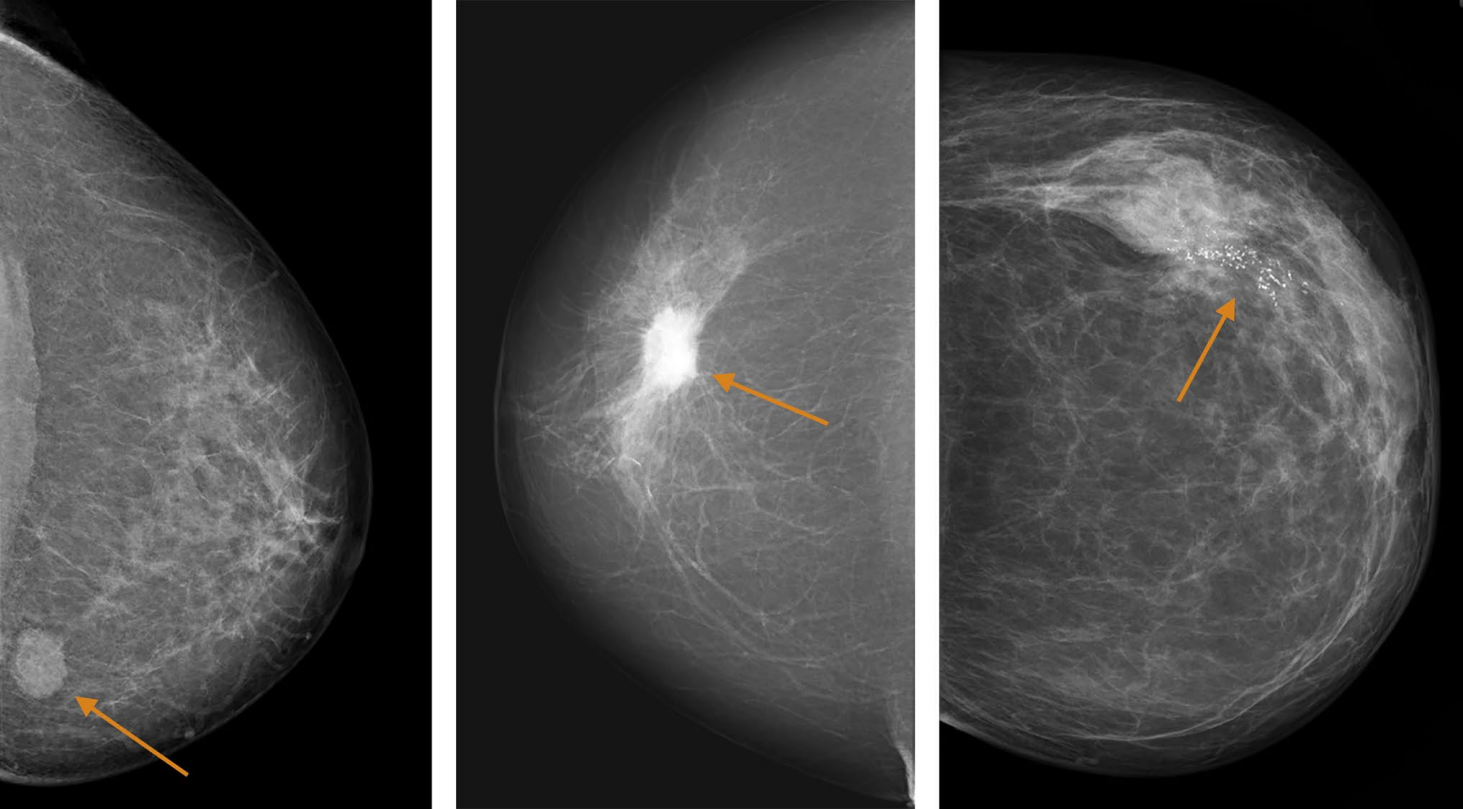
Mammographic tumour appearances. From left to right: distinct mass in the left breast of an 80-year-old woman, moderately dense breast. Spiculated mass in the right breast of 79-year-old woman, fat involuted breast. Calcifications in the left breast of a 77-year-old woman, moderately dense breast. All images acquired in the craniocaudal projection.

Information on the most dominant mammographic appearance of the tumour was obtained retrospectively. It should be noted that more than one appearance could have been readily visible and described in the original report, but only *the most dominant appearance* was recorded in the study protocol. Tumours were classified into the following comprehensive categories, based on the work by Luck et al.^13^: well-defined mass, partly ill-defined mass, ill-defined/diffuse mass, spiculated mass, comedo-type microcalcifications, non-specific calcifications, architectural distortion and asymmetrical density. For statistical analysis, these categories were converted into five larger categories: distinct mass (including well-defined and partly ill-defined tumours), ill-defined mass, spiculated mass, calcifications (including comedo-type and non-specific calcifications) and tissue abnormality (including the less frequent features architectural distortion and asymmetrical density). Some of the appearances are illustrated in Fig. 1.

#### Breast density

**Figure 2:**
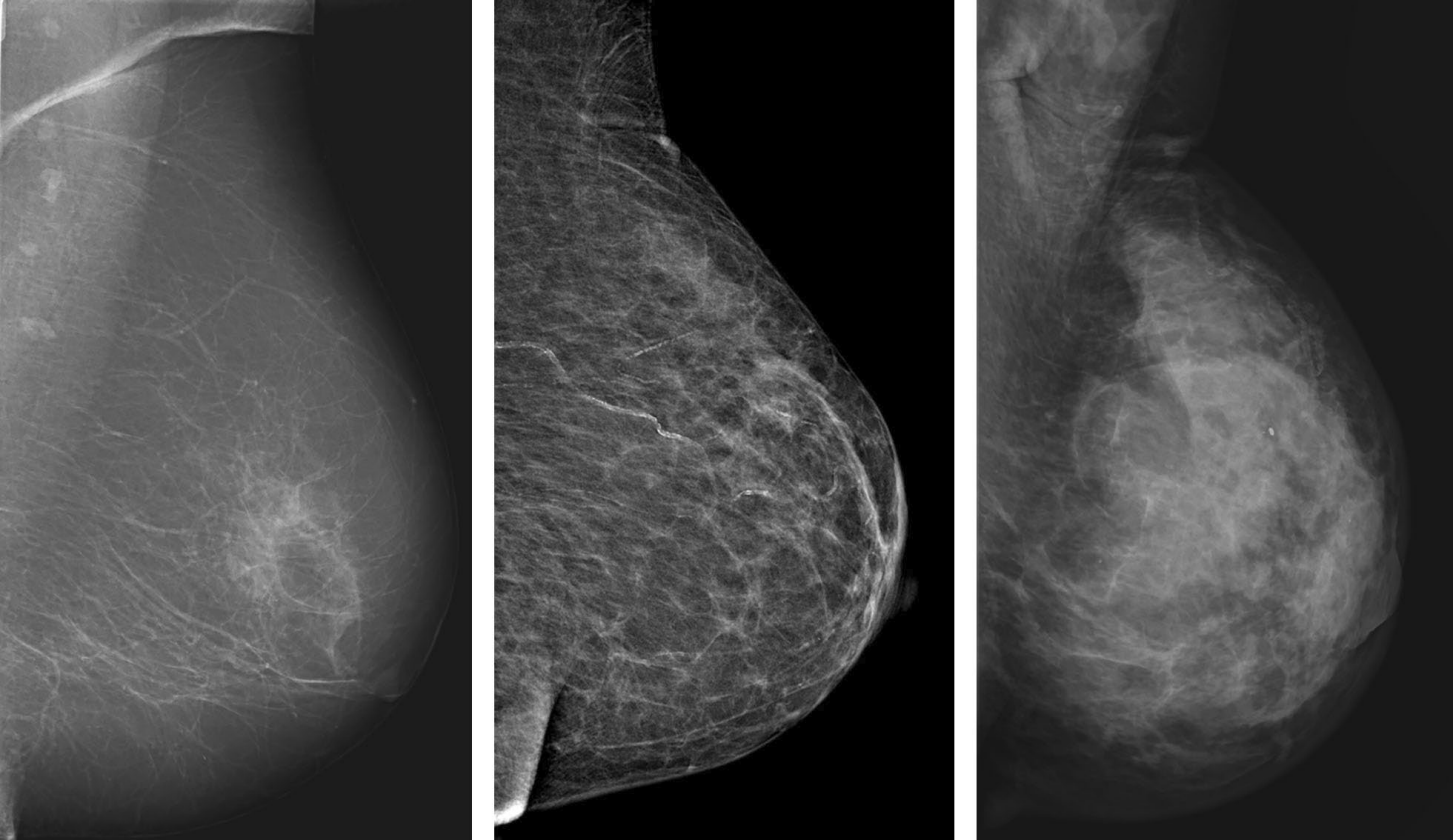
Breast density. From left to right: fat involuted breast. Moderately dense breast. Dense breast. All images acquired in the mediolateral oblique projection.

In the clinical setting at the Department of Breast Radiology in Malmö, breast density is divided into three groups: fat involuted, moderately dense and dense (Fig. 2). These three groups were employed in the present study. Fat involuted corresponds to Breast Imaging Reporting and Data System (BI-RADS) 4^14^ density score 1, moderately dense to BI-RADS 4 density score 2–3, and dense to BI-RADS 4 density score 4.

#### Mode of cancer detection

Method of cancer detection was divided into screening-detected or clinically detected, i.e. either detection via the breast cancer screening programme that started in Malmö in 1990, or detected clinically, due to a lump in the breast or other symptoms that caused the woman to seek medical attention. Thus, clinically detected tumours also include interval cancers, i.e. cancers diagnosed between screening episodes.

### Clinicopathological tumour factors

Information regarding clinicopathological factors was extracted from medical records and tissue microarray (TMA) evaluation. One TMA was constructed for breast cancers diagnosed between 1991 and 2004, and another one for breast cancers diagnosed between 2005 and 2007. For each tumour, two cores (0.6 mm in size 1991–2004 and 1.0 mm in size 2005–2007) from different representative areas of the tumour were collected for TMA construction. The details concerning the TMA construction have been described previously^15^.

#### Hormone receptors

Oestrogen receptor (ER) and progesterone receptor (PR) expression, based on immunohistochemical (IHC) staining, was extracted from TMA evaluation for the period 1991–2004, and from medical records for the period 2005–2014. IHC staining of 10% positive cells or less was considered negative, while values above 10% were interpreted as positive, in accordance with local clinical practice.

#### HER2

Information on HER2 status was retrieved from TMA evaluation from 1991 to 2007, and from medical records from 2008 to 2014. From 1991 to 2004, HER2 status was based solely on the IHC HercepTest^16^. Values of *0* and *1*+ were considered HER2 non-amplified (HER2−), and *3*+ amplified (HER2+), while *2*+ was entered as missing. In situ hybridization analysis was available from 2005 and onwards, and category *2*+ according to the HercepTest were from 2005 and onwards either categorized as amplified or non-amplified if conclusive in situ hybridization analysis had been performed, otherwise as missing, as described by Elebro et al.^15^.

#### Histological grade and tumour type

Breast cancers diagnosed from 1991 to 2004 were included in the first TMA and the histological grade (also Nottingham grade or Elston grade^17^) and histological type according to the World Health Organization classification was re-assessed by an experienced breast pathologist. For the period 2005–2014, this information was extracted from medical records^18^.

#### Ki67

Information on IHC proliferation marker Ki67 expression was collected during three time periods, from TMA assessment 1991–2004 and 2005–2007, and from medical records 2008–2014. Estimates of Ki67 expression can vary due to inter- and intra-observer variability, and due to variability in staining between pathology laboratories^19,20^. Ki67 status in the MDCS cohort varies during the long follow-up period of 13 years. Thus, using the same cut-off values for all three time periods could be misleading. To overcome this risk in the MDCS cohort, the values were divided into three equally sized groups for each time period^18^. The group with the lowest scores is denoted “low”, middle scores “intermediate”, and highest scores “high”.

#### Tumour size

Data on tumour size were collected from medical records during the entire follow-up period and were based on histopathological measurements. In multifocal breast cancer, the largest focus was used.

#### Surrogate molecular subtypes

The tumours were subsequently classified into surrogate molecular breast cancer subtypes in a modified St. Gallen 2013^21^ manner to facilitate the identification of different prognoses and characteristics. The mode of categorization was based on the local agreement of the southern Swedish breast cancer group, using the following subtypes: luminal A-like, luminal B-like, HER2+ subtype and TNBC^22^. All grade 1, ER positive (ER+) tumours were categorized as luminal A-like, regardless of Ki67 and PR status. Grade 2 ER+ tumours with low Ki67 expression, and grade 2 with intermediate Ki67 expression combined with PR positive (PR+) status were also classified as luminal A-like. All grade 3 ER+ tumours were considered to be luminal B-like, regardless of Ki67 and PR status. Grade 2 ER+ tumours with high Ki67 expression or intermediate Ki67 and PR− were classified as luminal B-like. All HER2+ tumours were considered HER2+ subtype, regardless of grade and hormone receptor status. ER−, PR− and HER2− tumours were considered TNBC.

### Statistical methods

Descriptive statistics, logistic regression, ordinal regression and multinomial logistic regression were carried out. The relation between mammographic appearance and ER, PR, and HER2 status was analysed with logistic regression, generating odds ratios (OR) and 95% confidence intervals (CI). To study the distribution of tumour appearance within the three categories of histological grade and Ki67, ordinal regression models were used, generating OR and 95% CI. The proportional odds assumption was assessed for each model, and if it holds, the OR:s can be interpreted as odds of being category 3 compared to the combined odds of being category 1 and 2. Potential associations between mammographic tumour appearance and surrogate molecular breast cancer subtypes were assessed through multinomial logistic regression, where distinct mass was set as a reference for the tumour appearances, and luminal A-like as a reference for the subtypes, yielding relative risk ratios (RRR) with 95% CI.

All statistical calculations were adjusted for breast density, mode of cancer detection, age at diagnosis and tumour size. Breast density was divided into three groups, and mode of detection into screening or clinical, as described in the *mammography* section above. Age at diagnosis was divided into quartiles. Tumour size was dichotomised into ≤ 20 mm and > 20 mm. In all the analyses, a p-value of less than 0.05 was considered statistically significant. Statistical analyses were performed with Stata version SE 14.2.

## Results

Table 1 provides information on the study population, including patient and tumour characteristics, in relation to mammographic appearance.

**Table 1:** Patient and tumour characteristics in relation to mammographic appearance.

	Distinct mass	Ill-defined mass	Spiculated	Calcifications	Tissue abnormality	Total
Age at diagnosis, median (range)	67 (48–89)	66 (45–91)	66 (49–91)	64 (48–83)	67 (48–89)	66 (45–91)
**Breast density**						
Fat involuted	57 (21.9)	30 (15.4)	83 (20.2)	4 (5.1)	6 (15.4)	180 (18.3)
Moderately dense	139 (53.5)	96 (49.2)	213 (52.0)	30 (38.5)	12 (30.7)	490 (49.9)
Dense	64 (24.6)	69 (35.4)	114 (27.8)	44 (56.4)	21 (53.9)	312 (31.8)
Missing	6	8	6	5	2	27
**Mode of detection**						
Screening detection	114 (43.0)	88 (43.4)	253 (60.8)	62 (74.7)	16 (39.0)	533 (52.9)
Clinical detection	151 (57.0)	115 (56.7)	163 (39.2)	21 (25.3)	25 (61.0)	475 (47.1)
Missing	1	0	0	0	0	1
**Tumour size (mm)**						
≤ 20	182 (73.7)	108 (55.7)	308 (75.3)	69 (85.2)	15 (45.5)	682 (70.8)
> 20	65 (26.3)	86 (44.3)	101 (24.7)	12 (14.8)	18 (54.5)	282 (29.2)
Missing	19	9	7	2	8	45
**Histological grade**						
1	56 (24.0)	34 (17.7)	127 (31.4)	22 (29.3)	11 (34.4)	250 (26.7)
2	91 (39.1)	95 (49.5)	210 (51.9)	32 (42.7)	14 (43.7)	442 (47.2)
3	86 (36.9)	63 (32.8)	68 (16.7)	21 (28.0)	7 (21.9)	245 (26.1)
Missing	33	11	11	8	9	72
**Histological type**						
Invasive ductal cancer	183 (76.6)	130 (67.0)	272 (67.3)	68 (85.0)	17 (53.1)	670 (70.6)
Invasive lobular cancer	12 (5.0)	56 (28.9)	92 (22.8)	7 (8.7)	13 (40.6)	180 (19.0)
Invasive tubular cancer	9 (3.8)	2 (1.0)	31 (7.7)	3 (3.7)	2 (6.3)	47 (5.0)
Invasive mucinous cancer	13 (5.4)	2 (1.0)	1 (0.3)	1 (1.3)	0 (0)	17 (1.8)
Other	22 (9.2)	4 (2.1)	8 (1.9)	1 (1.3)	0 (0)	35 (3.6)
Missing	27	9	12	3	9	60
**Oestrogen receptor**						
Negative	42 (18.4)	25 (13.9)	16 (4.1)	17 (25.8)	2 (6.2)	102 (11.4)
Positive	186 (81.6)	155 (86.1)	372 (95.9)	49 (74.2)	30 (93.8)	792 (88.6)
Missing	38	23	28	17	9	115
**Progesterone receptor**						
Negative	96 (43.0)	77 (44.5)	115 (30.9)	33 (51.6)	17 (54.8)	338 (39.2)
Positive	127 (57.0)	96 (55.5)	257 (69.1)	31 (48.4)	14 (45.2)	525 (60.8)
Missing	43	30	44	19	10	146
**HER2**						
Negative	199 (90.5)	140 (84.9)	342 (94.5)	49 (84.5)	29 (93.6)	759 (90.8)
Positive	21 (9.5)	25 (15.1)	20 (5.5)	9 (15.5)	2 (6.4)	77 (9.2)
Missing	46	38	54	25	10	173
**Ki67**						
Low	55 (27.9)	49 (32.5)	146 (45.3)	21 (37.5)	15 (50.0)	286 (37.8)
Intermediate	67 (34.0)	45 (29.8)	110 (34.2)	16 (28.6)	6 (20.0)	244 (32.3)
High	75 (38.1)	57 (37.7)	66 (20.5)	19 (33.9)	9 (30.0)	226 (29.9)
Missing	69	52	94	27	11	253
**Breast cancer subtype**						
Luminal A-like	95 (46.8)	67 (44.1)	215 (67.4)	23 (45.1)	19 (63.3)	419 (55.5)
Luminal B-like	52 (25.6)	43 (28.3)	74 (23.2)	12 (23.5)	7 (23.3)	188 (24.9)
HER2+ subtype	21 (10.3)	25 (16.5)	20 (6.3)	9 (17.7)	2 (6.7)	77 (10.2)
Triple-negative breast cancer	35 (17.2)	17 (11.1)	10 (3.1)	7 (13.7)	2 (6.7)	71 (9.4)
Missing	63	51	97	32	11	254

Data presented as counts (percent) unless otherwise stated.

### Tumour appearance in relation to clinicopathological factors

Ill-defined tumours, spiculated tumours and tumours presenting as tissue abnormality were more likely to be ER+ than ER−, as compared to a distinct mass, according to logistic regression analysis, with an adjusted OR (OR_adj)_ of 2.0 (CI 1.1–3.6), 6.0 (CI 3.2–11.2) and 4.4 (CI 1.0–19.6), respectively (Table 2). Furthermore, spiculated tumours were more likely to be PR+ than PR− compared to a distinct mass, with an OR_adj_ of 1.7 (CI 1.2–2.5) (Table 2). We found no statistical evidence for an association between mammographic appearance and HER2 status (Table 2). However, ill-defined masses and tumours presenting as calcifications were slightly more often HER2+ than were the other tumour features in terms of distribution. According to ordinal regression analysis, the odds of spiculated tumours, compared to distinct masses, to be histological grade 3, compared to the combined categories of grade 1 and 2, were 50% lower, OR_adj_ 0.5 (CI 0.4–0.7), given that all the other variables in the model were kept constant (Table 3). Tumours presenting as tissue abnormalities were also more likely to be of grade 1 or 2, rather than grade 3, compared to distinct masses, with an OR_adj_ of 0.3 (CI 0.1–0.6). Spiculated tumours were more likely to exhibit lower Ki67 expression compared to distinct masses, with an OR_adj_ of high Ki67 compared to the combined categories of low and intermediate Ki67 of 0.5 (CI 0.3–0.6). In addition, tissue abnormality had lower odds of being in the high Ki67 expression category as compared to distinct masses, with an OR_adj_ of 0.3 (CI 0.2–0.8) (Table 3).

**Table 2:** Mammographic appearance in relation to oestrogen receptor (ER), progesterone receptor (PR) and human epidermal growth factor 2 (HER2) status.

Mammographic appearance	ER−	ER+	OR (95% CI)	p-value	OR_adj_* (95% CI)	p-value
	n (%)					
				< 0.001		< 0.001
Distinct mass	42 (18.4)	186 (81.6)	1.0		1.0	
Ill-defined mass	25 (13.9)	155 (86.1)	1.4 (0.8–2.4)	0.221	2.0 (1.1–3.6)	0.022
Spiculated	16 (4.1)	372 (95.9)	5.2 (2.9–9.6)	< 0.001	6.0 (3.2–11.2)	< 0.001
Calcifications	17 (25.8)	49 (74.2)	0.7 (0.3–1.2)	0.192	0.6 (0.3–1.3)	0.191
Tissue abnormality	2 (6.3)	30 (93.8)	3.4 (0.8–14.7)	0.104	4.4 (1.0–19.6)	0.054
Observations			894		867	

Mammographic appearance	PR−	PR+	OR (95% CI)	p-value	OR_adj_* (95% CI)	p-value
	n (%)					
				< 0.001		0.007
Distinct mass	96 (43.1)	127 (56.9)	1.0		1.0	
Ill-defined mass	77 (44.5)	96 (55.5)	0.9 (0.6–1.4)	0.772	1.1 (0.7–1.7)	0.648
Spiculated	115 (30.9)	257 (69.1)	1.7 (1.2–2.4)	0.003	1.7 (1.2–2.5)	0.003
Calcifications	33 (51.6)	31 (48.4)	0.7 (0.4–1.2)	0.229	0.8 (0.5–1.5)	0.543
Tissue abnormality	17 (54.8)	14 (45.2)	0.6 (0.3–1.3)	0.219	0.9 (0.4–2.1)	0.826
Observations			863		839	

Mammographic appearance	HER2−	HER2+	OR (95% CI)	p-value	OR_adj_* (95% CI)	p-value
	n (%)					
				0.005		0.021
Distinct mass	199 (90.5)	21 (9.5)	1.0		1.0	
Ill-defined mass	140 (84.8)	25 (15.2)	1.7 (0.9–3.1)	0.096	1.5 (0.8 – 3.0)	0.208
Spiculated	342 (94.5)	20 (5.5)	0.6 (0.3–1.0)	0.069	0.6 (0.3–1.1)	0.107
Calcifications	49 (84.5)	9 (15.5)	1.7 (0.8–4.0)	0.197	2.0 (0.8–5.0)	0.134
Tissue abnormality	29 (93.5)	2 (6.5)	0.7 (0.1–2.9)	0.597	0.7 (0.1–3.1)	0.621
Observations			836		814	

*Adjusted for age (categorical), tumour size, mode of detection and breast density.

**Table 3:** Mammographic appearance in relation to histological grade and Ki67 expression.

Mammographic appearance	Grade 1	Grade 2	Grade 3	OR (95% CI)	p-value	OR_adj_* (95% CI)	p-value
	n (%)						
					< 0.001		< 0.001
Distinct mass	56 (24.0)	91 (39.1)	86 (36.9)	1.0		1.0	
Ill-defined mass	34 (17.7)	95 (49.5)	63 (32.8)	1.0 (0.7–1.5)	0.861	0.8 (0.6–1.2)	0.339
Spiculated	127 (31.4)	210 (51.9)	68 (16.7)	0.5 (0.4–0.7)	< 0.001	0.5 (0.4–0.7)	< 0.001
Calcifications	22 (29.3)	32 (42.7)	21 (28.0)	0.7 (0.4–1.1)	0.123	0.9 (0.5–1.5)	0.602
Tissue abnormality	11 (34.4)	14 (43.8)	7 (21.8)	0.5 (0.2–1.0)	0.054	0.3 (0.1–0.6)	0.002
Observations				937		912	

Mammographic appearance	Low Ki67	Intermediate Ki67	High Ki67	OR (95% CI)	p-value	OR_adj_* (95% CI)	p-value
	n (%)						
					< 0.001		< 0.001
Distinct mass	55 (27.9)	67 (34.0)	75 (38.1)	1.0		1.0	
Ill-defined mass	49 (32.4)	45 (29.8)	57 (37.8)	0.9 (0.6–1.3)	0.600	0.8 (0.5–1.1)	0.175
Spiculated	146 (45.3)	110 (34.2)	66 (20.5)	0.5 (0.3–0.6)	< 0.001	0.5 (0.3–0.6)	< 0.001
Calcifications	21 (37.5)	16 (28.6)	19 (33.9)	0.7 (0.4–1.3)	0.217	0.9 (0.5–1.6)	0.708
Tissue abnormality	15 (50.0)	6 (20.0)	9 (30.0)	0.5 (0.2–1.0)	0.055	0.3 (0.2–0.8)	0.009
Observations				756		737	

*Adjusted for age (categorical), tumour size, mode of detection and breast density.

### Tumour appearance in relation to surrogate molecular subtypes

The frequencies of the mammographic appearances within the four breast cancer subtypes are given in Table 4. It was found to be more likely that an ill-defined mass would be of luminal A-like subtype than TNBC, than would a distinct mass, with an RRR_adj_ of 0.5 (CI 0.2–0.9), according to multinomial logistic regression analysis (Table 5). The relative risk of a spiculated tumour, compared to a distinct mass, being luminal B-like, HER2+ subtype or TNBC, compared to luminal A-like was lower: RRR_adj_ 0.6 (CI 0.4–1.0), 0.4 (CI 0.2–0.8) and 0.1 (0.1–0.3), respectively. Or expressed more generally, it is more likely that a spiculated tumour will be luminal A-like than luminal B-like, HER2+ subtype or TNBC. No evidence was found of any association between molecular subtype and tumours presenting as calcifications. Moreover, tumours presenting as tissue abnormality were less likely to be TNBC than luminal A-like, with an RRR_adj_ of 0.2 (CI 0.0–0.8) (Table 5).

**Table 4:** Mammographic appearance and frequencies of molecular subtypes.

Molecular subtype	Luminal A-like	Luminal B-like	HER2+ subtype	Triple-negative breast cancer
Mammographic appearance	n (%)			
Distinct mass	95 (46.8)	52 (25.6)	21 (10.3)	35 (17.3)
Ill-defined mass	67 (44.1)	43 (28.2)	25 (16.5)	17 (11.2)
Spiculated	215 (67.4)	74 (23.2)	20 (6.3)	10 (3.1)
Calcifications	23 (45.1)	12 (23.5)	9 (17.7)	7 (13.7)
Tissue abnormality	19 (63.3)	7 (23.3)	2 (6.7)	2 (6.7)

**Table 5:** Mammographic appearance in relation to molecular subtypes.

Molecular subtype	Luminal A-like	Luminal B-like	p-value	Human epidermal growth factor 2 (HER2)+ subtype	p-value	Triple negative breast cancer	p-value
	Reference	RRR (95% CI)		RRR (95% CI)		RRR (95% CI)	
**Mammographic appearance**							< 0.001
Distinct mass		1.0		1.0		1.0	
Ill-defined mass		1.2 (0.7–2.0)	0.541	1.7 (0.9–3.3)	0.119	0.7 (0.4–1.3)	0.267
Spiculated		0.6 (0.4–1.0)	0.034	0.4 (0.2–0.8)	0.010	0.1 (0.1–0.3)	< 0.001
Calcifications		1.0 (0.4–2.1)	0.904	1.8 (0.7–4.4)	0.216	0.8 (0.3–2.1)	0.687
Tissue abnormality		0.7 (0.3–1.7)	0.404	0.5 (0.1–2.2)	0.342	0.3 (0.1–1.3)	0.342
Observations		755					

Molecular subtype	Luminal A-like	Luminal B-like	p-value	HER2+ subtype	p-value	Triple negative breast cancer	p-value
	Reference	RRR_adj_* (95% CI)		RRR_adj_* (95% CI)		RRR_adj_* (95% CI)	
**Mammographic appearance**							< 0.001
Distinct mass		1.0		1.0		1.0	
Ill-defined mass		0.9 (0.5–1.6)	0.844	1.4 (0.7–2.8)	0.387	0.5 (0.2–0.9)	0.033
Spiculated		0.6 (0.4–1.0)	0.061	0.4 (0.2–0.8)	0.015	0.1 (0.1–0.3)	< 0.001
Calcifications		1.4 (0.6–3.1)	0.464	2.4 (0.9–6.5)	0.085	0.9 (0.3–2.5)	0.861
Tissue abnormality		0.4 (0.1–1.1)	0.069	0.4 (0.1–1.7)	0.197	0.2 (0.0–0.8)	0.023
Observations		737					

*Adjusted for age (categorical), tumour size, mode of detection and breast density.

## Discussion

In this large study on 1116 cases of incident breast cancer, we found strong evidence supporting that established clinicopathological factors and surrogate molecular subtypes differ in regard to mammographic tumour appearance. A particularly interesting result was that all the methods of statistical analysis indicated an association between spiculation and favourable tumour characteristics.

The finding that spiculated tumours are more often ER+ and PR+ is in agreement with several previous studies^23–27^. However, the cut-off values for hormone receptor positivity and tumour appearance categorization differ between some studies^23,26^. In agreement with several other studies, we found spiculation to be associated with lower histological grade^28–30^ and lower Ki67 values^23,26^. A previous study^31^, in which a different categorization of mammographic appearance was used, revealed an association between higher histological grade and spiculation with calcifications. However, in that study, the association with higher histological grade was even stronger in non-spiculated tumours, both with and without calcifications. Despite some methodological differences between our study and previous studies, it is clear that spiculation is indicative of favourable tumour characteristics. On the cellular level, the spicules of malignant tumours can represent tumour infiltration, a desmoplastic response in the adjacent stroma or periductal fibrosis^32^. However, the link between these features and the favourable characteristics of spiculated tumours is not clear. Future studies on spiculation and the molecular mammary microenvironment may increase this knowledge.

According to our findings, ill-defined masses were also more likely to be ER+, however, we could find no evidence in the literature supporting or contradicting this finding. This could be because the category ill-defined mass is seldom used, and when it is, often with other outcomes^13,29,33^ than those considered in the present study. Regarding HER2, we found no evidence of any association with tumour appearance. Previous studies have shown an association between spiculation and HER2−^23,26^ as well as calcifications and HER2+^9,34–36^. In terms of frequency, the majority of spiculated tumours in our study were HER2− (94.5%), and calcifications were more often HER2+ (15.5%). Hence, our results point in the same direction, although with weak statistical support. Tumours presenting as tissue abnormality are more likely to be ER+, of lower histologic grade and lower Ki67 expression. The number of observations in this group is however small, making it difficult to draw any reliable conclusions.

Information on surrogate molecular subtypes is essential in breast cancer management. These were therefore included in this study to refine our analyses and to make the results more clinically comprehensible. Spiculated tumours were more commonly luminal A-like subtype, which is in line with the findings of three previous studies^26,33,37^, one of which^37^ highlighted women under the age of 40. Breast density, which is higher in younger women, is an interesting factor that can affect tumour appearance to some degree. By adjusting for breast density, we were able to show that the association between spiculation and luminal A-like subtype persists, regardless of density. Tamaki et al.^38^ found that masses classified as having indistinct margins on mammography, according to BI-RADS, were more often HER2+ subtype or TNBC than luminal type cancers. This is in contrast to our finding that ill-defined masses are more often luminal A-like than TNBC. The reason for this discrepancy is not known, but could be due to differences in mammographic categorization and population sampling. In the present study, the appearance was extracted from the original radiology report, and BI-RADS is not used in clinical practice at our department. In addition, their population was younger, with a median age of 50 years (range 27–89 years), compared to a median age of 66 years (range 45–91 years) in the present study. Associations between various types of calcifications and HER+ subtype have been described previously^36,39,40^. However, we found no evidence of this in our study. In order to increase group sizes, we combined all types of calcifications into one group, and it was therefore not possible to study differences between various types of calcifications. Several other studies have reported that TNBC is associated with a mammographic mass and can be mistaken for a benign breast lesion^10,41–44^. It is therefore of the utmost importance to investigate these lesions carefully. In terms of frequency, a large proportion of the distinct masses in our study constituted TNBC (17.3%), as opposed to spiculated tumours, of which only a few (3.1%) were TNBC (Table 4). However, a potential association between TNBC and distinct mass in our material cannot be directly identified due to the setup of statistical analysis.

Some issues require consideration. Firstly, this was a retrospective observational study performed at a single department. Secondly, the women with breast cancer in the MDCS tend to be of ethnic Swedish descent, and with a higher level of education than the average population^45^, which could limit the representativeness of the findings. However, the clinicopathological factors studied were distributed as expected in routine clinical practice. Hence, we believe there is a low risk of selection bias, and that the internal comparisons should not be affected. Thirdly, the use of TMA for evaluation of clinicopathological parameters is worth considering as there is always a risk that the cores obtained for TMA do not reflect the original tumour and its potential heterogeneity correctly. However, this risk is reduced by obtaining two cores from different areas of each tumour, an approach that has been shown to be highly representative in breast cancer^46^. Furthermore, agreement between TMA assessment and clinical records has been shown to be high^47^. Fourthly, digital mammography was implemented in 2004, and therefore both analogue and digital images were included in this study. However, this has been shown not to influence screening performance^48^. Finally, a wide range of mammographic appearances is used in the literature^6,10,26^, which leads to difficulties when comparing studies. Also, we only considered the most dominant appearance, while others have considered combinations of appearances. Nevertheless, we believe our results to be generalizable, as spiculated appearance is often treated as a separate entity in the literature. This study confirms the clinical notion that breast tumours with a spiculated appearance on mammography have more favourable characteristics and hence the prognosis is potentially better. Survival was not an endpoint in this study, but is planned for a future study. Overdiagnosis of slow-growing tumours that would perhaps not have led to breast cancer morbidity or mortality is a well-known issue in breast cancer screening. In this study, spiculated tumours were more frequent among screening-detected than clinically detected tumours, which may indicate that some of these tumours were so-called over-diagnosed cancers.

In conclusion, this study provides strong statistical evidence of several associations between mammographic tumour appearance and clinicopathological factors, including molecular subtypes. In particular, the results consistently indicate favourable characteristics of spiculated tumours. Defining associations between the mammographic tumour appearance and the clinicopathological outcome may aid in characterizing breast cancer already from the initial mammogram, which could potentially contribute to more individualized breast cancer treatment in the future.

## Data Availability

The data supporting the findings of this study are available from the Malmö Cohorts. However, restrictions apply to the availability of the data, which were used under license for the current study, and are not publicly available. For more information visit the Malmö Cohorts webpage: https://www.malmo-kohorter.lu.se/English.

## References

[1] Eccles SA (2013). Critical research gaps and translational priorities for the successful prevention and treatment of breast cancer. Breast Cancer Res..

[2] Cherel P, Becette V, Hagay C (2005). Stellate images: Anatomic and radiologic correlations. Eur. J. Radiol..

[3] Berment H, Becette V, Mohallem M, Ferreira F, Cherel P (2014). Masses in mammography: What are the underlying anatomopathological lesions?. Diagn. Interv. Imaging.

[4] Tabar L (2004). Mammographic tumor features can predict long-term outcomes reliably in women with 1–14-mm invasive breast carcinoma. Cancer.

[5] Sartor H (2015). Do mammographic tumor features in breast cancer relate to breast density and invasiveness, tumor size, and axillary lymph node involvement?. Acta Radiol. (Stockholm, Sweden).

[6] Alexander MC, Yankaskas BC, Biesemier KW (2006). Association of stellate mammographic pattern with survival in small invasive breast tumors. Am. J. Roentgenol..

[7] Andersson I (2008). Breast tomosynthesis and digital mammography: A comparison of breast cancer visibility and BIRADS classification in a population of cancers with subtle mammographic findings. Eur. Radiol..

[8] Curigliano G (2017). De-escalating and escalating treatments for early-stage breast cancer: The St. Gallen International Expert Consensus Conference on the Primary Therapy of Early Breast Cancer 2017. Ann. Oncol..

[9] Radenkovic S (2014). HER2-positive breast cancer patients: Correlation between mammographic and pathological findings. Radiat. Prot. Dosimetry.

[10] Killelea BK (2013). Is there a correlation between breast cancer molecular subtype using receptors as surrogates and mammographic appearance?. Ann. Surg. Oncol..

[11] Manjer J, Elmstahl S, Janzon L, Berglund G (2002). Invitation to a population-based cohort study: Differences between subjects recruited using various strategies. Scand. J. Public Health.

[12] Berglund G, Elmstahl S, Janzon L, Larsson SA (1993). The Malmo Diet and Cancer Study. Design and feasibility. J. Internal Med..

[13] Luck AA (2008). Breast carcinoma with basal phenotype: Mammographic findings. Am. J. Roentgenol..

[14] Sickles E, D’Orsi CJ, Bassett LW (2013). ACR BI-RADS® Atlas, Breast Imaging Reporting and Data System.

[15] Elebro K, Butt S, Dorkhan M, Jernstrom H, Borgquist S (2014). Age at first childbirth and oral contraceptive use are associated with risk of androgen receptor-negative breast cancer: The Malmo Diet and Cancer Cohort. Cancer Causes Control.

[16] Dowsett M (2003). Correlation between immunohistochemistry (HercepTest) and fluorescence in situ hybridization (FISH) for HER-2 in 426 breast carcinomas from 37 centres. J. Pathol..

[17] Elston CW, Ellis IO (1991). Pathological prognostic factors in breast cancer. I. The value of histological grade in breast cancer: Experience from a large study with long-term follow-up. Histopathology.

[18] Huss L (2019). Vitamin D receptor expression in invasive breast tumors and breast cancer survival. Breast Cancer Res..

[19] Dowsett M (2011). Assessment of Ki67 in breast cancer: Recommendations from the International Ki67 in Breast Cancer working group. J. Natl. Cancer Inst..

[20] Focke CM (2017). Interlaboratory variability of Ki67 staining in breast cancer. Eur. J. Cancer (Oxford, England: 1990).

[21] Goldhirsch A (2013). Personalizing the treatment of women with early breast cancer: Highlights of the St Gallen International Expert Consensus on the Primary Therapy of Early Breast Cancer 2013. Ann. Oncol..

[22] Guidelines for surgical and oncologic treatment of breast cancer—regional adaption of the national treatment guidelines, Region West and South (in Swedish). https://www.cancercentrum.se/globalassets/cancerdiagnoser/brost/syd/sydsvenska-brostcancergruppens-lathund-170401-180331.pdf (2017).

[23] Jiang L (2011). Mammographic features are associated with clinicopathological characteristics in invasive breast cancer. Anticancer Res..

[24] Broberg A, Glas U, Gustafsson SA, Hellstrom L, Somell A (1983). Relationship between mammographic pattern and estrogen receptor content in breast cancer. Breast Cancer Res. Treat..

[25] Ildefonso C (2008). The mammographic appearance of breast carcinomas of invasive ductal type: Relationship with clinicopathological parameters, biological features and prognosis. Eur. J. Obstet. Gynecol. Reprod. Biol..

[26] Liu S (2016). Is there a correlation between the presence of a spiculated mass on mammogram and luminal A subtype breast cancer?. Korean J. Radiol..

[27] Nielsen NS, Poulsen HS (1985). Relation between mammographic findings and hormonal receptor content in breast cancer. Am. J. Roentgenol..

[28] De Nunzio MC (1997). Correlations between the mammographic features of screen detected invasive breast cancer and pathological prognostic factors. Breast.

[29] Evans AJ, Pinder SE, James JJ, Ellis IO, Cornford E (2006). Is mammographic spiculation an independent, good prognostic factor in screening-detected invasive breast cancer?. Am. J. Roentgenol..

[30] Lamb PM, Perry NM, Vinnicombe SJ, Wells CA (2000). Correlation between ultrasound characteristics, mammographic findings and histological grade in patients with invasive ductal carcinoma of the breast. Clin. Radiol..

[31] Shin HJ (2011). Correlation between mammographic and sonographic findings and prognostic factors in patients with node-negative invasive breast cancer. Br. J. Radiol..

[32] Franquet T, De Miguel C, Cozcolluela R, Donoso L (1993). Spiculated lesions of the breast: Mammographic–pathologic correlation. Radiogr. Rev. Publ. Radiol. Soc. N. Am. Inc.

[33] Taneja S (2008). The mammographic correlations of a new immunohistochemical classification of invasive breast cancer. Clin. Radiol..

[34] Gajdos C (2002). Mammographic appearance of nonpalpable breast cancer reflects pathologic characteristics. Ann. Surg..

[35] Sun SS, Zhang B, Zhao HM, Cao XC (2014). Association between mammographic features and clinicopathological characteristics in invasive ductal carcinoma of breast cancer. Mol. Clin. Oncol..

[36] Elias SG (2014). Imaging features of HER2 overexpression in breast cancer: A systematic review and meta-analysis. Cancer epidemiol. Biomark. Prev..

[37] Bullier B (2013). Imaging features of sporadic breast cancer in women under 40 years old: 97 cases. Eur. Radiol..

[38] Tamaki K (2011). Correlation between mammographic findings and corresponding histopathology: Potential predictors for biological characteristics of breast diseases. Cancer Sci..

[39] Bare M (2015). Mammographic and clinical characteristics of different phenotypes of screen-detected and interval breast cancers in a nationwide screening program. Breast Cancer Res. Treat..

[40] Cen D (2017). BI-RADS 3–5 microcalcifications can preoperatively predict breast cancer HER2 and Luminal a molecular subtype. Oncotarget.

[41] Boisserie-Lacroix M (2013). Triple-negative breast cancers: Associations between imaging and pathological findings for triple-negative tumors compared with hormone receptor-positive/human epidermal growth factor receptor-2-negative breast cancers. Oncologist.

[42] Dogan BE, Turnbull LW (2012). Imaging of triple-negative breast cancer. Ann. Oncol..

[43] Gao B (2014). Mammographic and clinicopathological features of triple-negative breast cancer. Br. J. Radiol..

[44] Kim MY, Choi N (2013). Mammographic and ultrasonographic features of triple-negative breast cancer: A comparison with other breast cancer subtypes. Acta Radiol. (Stockholm, Sweden: 1987).

[45] Manjer J (2001). The Malmo Diet and Cancer Study: Representativity, cancer incidence and mortality in participants and non-participants. Eur. J. Cancer prev..

[46] Camp RL, Charette LA, Rimm DL (2000). Validation of tissue microarray technology in breast carcinoma. Lab. Investig. J. Tech. Methods Pathol..

[47] Allott EH (2016). Intratumoral heterogeneity as a source of discordance in breast cancer biomarker classification. Breast Cancer Res..

[48] Song SY (2019). Comparison of digital and screen-film mammography for breast-cancer screening: A systematic review and meta-analysis. J. Breast Cancer.

